# Genotype- and tissue-specific miRNA profiles and their targets in three alfalfa (*Medicago sativa L*) genotypes

**DOI:** 10.1186/s12864-018-5280-y

**Published:** 2018-12-31

**Authors:** Robert Pokoo, Shuchao Ren, Qingyi Wang, Christy M. Motes, Timothy D. Hernandez, Sayvan Ahmadi, Maria J. Monteros, Yun Zheng, Ramanjulu Sunkar

**Affiliations:** 1 Department of Biochemistry and Molecular Biology, Oklahoma State University, Stillwater, OK 74078 USA; 2 Institute of Primate Translational Medicine, Kunming University of Science and Technology, 727 South Jingming Road, Kunming, 650500 Yunnan China; 3 Noble Research Institute, Ardmore, OK 73401 USA; 4 Faculty of Information Engineering and Automation, Kunming University of Science and Technology, 727 South Jingming Road, Kunming, 650500 Yunnan China

## Abstract

**Background:**

Alfalfa (*Medicago sativa* L.) is a forage legume with significant agricultural value worldwide. MicroRNAs (miRNAs) are key components of post-transcriptional gene regulation and essentially regulate many aspects of plant growth and development. Although miRNAs were reported in alfalfa, their expression profiles in different tissues and the discovery of novel miRNAs as well as their targets have not been described in this plant species.

**Results:**

To identify tissue-specific miRNA profiles in whole plants, shoots and roots of three different alfalfa genotypes (Altet-4, NECS-141and NF08ALF06) were used. Small RNA libraries were generated and sequenced using a high-throughput sequencing platform. Analysis of these libraries enabled identification of100 miRNA families; 21 of them belong to the highly conserved families while the remaining 79 families are conserved at the minimum between *M. sativa* and the model legume and close relative*, M. truncatula*. The profiles of the six abundantly expressed miRNA families (miR156, miR159, miR166, miR319, miR396 and miR398) were relatively similar between the whole plants, roots and shoots of these three alfalfa genotypes. In contrast, robust differences between shoots and roots for miR160 and miR408 levels were evident, and their expression was more abundant in the shoots. Additionally, 17 novel miRNAs were identified and the relative abundance of some of these differed between tissue types. Further, the generation and analysis of degradome libraries from the three alfalfa genotypes enabled confirmation of 69 genes as targets for 31 miRNA families in alfalfa.

**Conclusions:**

The miRNA profiles revealed both similarities and differences in the expression profiles between tissues within a genotype as well as between the genotypes. Among the highly conserved miRNA families, miR166 was the most abundantly expressed in almost all tissues from the three genotypes. The identification of conserved and novel miRNAs as well as their targets in different tissues of multiple genotypes increased our understanding of miRNA-mediated gene regulation in alfalfa and could provide valuable insights for practical research and plant improvement applications in alfalfa and related legume species.

## Introduction

Alfalfa (*Medicago sativa* L.) is an important forage legume species with global adaptation, high forage quality and the capacity for harvesting biomass multiple times during the growing season. Alfalfa is an autotetraploid (2n = 4x = 32), perennial outcrossing species with high levels of genetic diversity in cultivated and non-cultivated populations. Besides its use as a forage, alfalfa also has potential crop for biofuel production [1]. Alfalfa has the capacity for symbiotic nitrogen fixation and can also contribute to reduce soil erosion [2, 3].

Endogenous non-coding RNAs of approximately 21–22 nucleotides represent plant miRNAs that silence gene expression by binding to complementary sequences of its target mRNA at the post-transcriptional level. Such targeting results in mRNA cleavage and degradation or repression of translation, with the former being more prevalent in plants [4–7]. The miRNA analyses in different plant species highlight the important regulatory roles of miRNAs in multiple organs (roots, stems, leaves and flowers), differentiation and development, leaf polarity, transition from juvenile to vegetative stages and vegetative to reproductive phases, and regulation of plant responses to biotic and abiotic stresses [8–10].

Several investigations have shown that plant miRNAs can be classified into conserved and novel lineage- or species-specific miRNAs. Conserved miRNAs and their corresponding target genes are commonly found in all or most angiosperms, with some also being described in gymnosperms as well as primitive land plants such as ferns [11, 12]. However, miRNA analysis in several legumes including *M. truncatula*, soybean (*Glycine max* L), chickpea (*Cicer arietinum* L.), common bean (*Phaseolus vulgaris*), and *Lotus japonicus* indicate the presence of miRNAs that seem to be specific to certain legumes and there could have important gene regulatory roles [13–19]. Although recent attempts were made to report miRNAs from alfalfa (*M. sativa*) [20–22], these do not include the discovery of novel miRNAs, and most importantly, the miRNA target genes have not been confirmed in this legume species. Understanding miRNAs and their target gene regulation in various tissues can provide further insights into the miRNA target networks operating in a tissue-specific manner in alfalfa.

In order to identify conserved miRNAs as well as novel miRNAs from alfalfa, we constructed and sequenced small RNA libraries from whole clonally propagated plants, roots and shoots of three alfalfa genotypes (Altet-4, NECS-141 and NF08ALF06). The sequenced reads were mapped to known miRNAs in *M. truncatula*, deposited in the miRBase to identify and annotate the miRNAs in alfalfa. Degradome libraries were constructed and sequenced from these three genotypes to characterize the miRNA gene targets.

## Materials and methods

### Plant materials and growth conditions

Three alfalfa genotypes NECS-141, Altet-4 and NF08ALF06 were evaluated in this study. NECS-141 is the genotype being used to sequence the tetraploid alfalfa genome [23]. Altet-4 is an aluminum tolerant genotype used to develop a mapping population [24]. NF08ALF06 is a commercial breeding line with good agronomic performance (Forage Genetics International). The three alfalfa genotypes (NECS-141, Altet-4 and NF08ALF06) were clonally propagated and grown in tissue culture. After 13 d of growth in rooting media, these were transferred to medium at pH 7 for 96 h as previously described [25]. The rooting media contains 0.55 g/L Murashige & Skoog Basal Medium with Vitamins (PhytoTechnology #M519), 1 ml Plant Preservative Mixture, PPM (PhytoTechnology), adjust the pH to 5.8, and add 12 g/L Gelzan. The plants were placed in a Conviron growth chamber (24 °C, 18 h /6 h day/night cycle, 100 μmol light intensity) for root development and growth. An additional 20 clonally propagated plants of these genotypes were grown in a Conviron growth chamber as previously described and used to evaluate the tissue-specific expression of the miRNAs. Tissue samples were harvested and immediately flash frozen in liquid nitrogen and stored at − 80 °C.

### Small RNA library construction and sequencing

Total RNA was isolated from the whole plants, roots and shoots of three alfalfa genotypes using TRIzol ® Reagent (Invitrogen), according to the manufacturer’s instructions. The quality of total RNA was monitored on 1% agarose gel and their concentrations were measured using Nanodrop spectrophotometer. Small RNA libraries were generated as described previously [26] by following the protocol described for the Illumina Truseq® Small RNA Preparation kit (Illumina, San Diego, USA). Briefly, 1 μg total RNA per sample was ligated sequentially with 5′ and 3’ RNA adaptors. The ligated products were converted into cDNAs and then amplified using PCR. The amplified products were sequenced using an Illumina Hiseq® Analyzer.

### Identification of conserved and novel miRNAs

The raw sequencing reads were processed as follows: adaptor sequences were trimmed off from the raw reads to obtain small RNAs. These reads were then mapped to ribosomal RNA (rRNA), transfer RNA (tRNA), small nuclear RNAs (snRNA), and the aligned and mapped reads were not used for further analysis. The remaining reads were aligned to miRBase v 20 [27] to identify miRNAs in *M. sativa*. The reads with 100% sequence identity were designated as conserved miRNA homologs. To identify novel miRNAs, the presence of the miRNA-star (miRNA*) sequences coupled with the predictable hairpin-like structure for the precursor sequences were used.

### Degradome library construction and analyses

Degradome libraries from the alfalfa genotypes NECS-141, Altet-4 and NF08ALF06 were constructed as previously described to identify potential target mRNAs [28]. Briefly, the cleaved 5′ monophosphate containing polyadenylated mRNA fragments were ligated to an RNA oligo-nucleotide adapter containing *MmeI* recognition site at its 3′ end. The ligated products were converted into cDNA using reverse transcriptase and the product was amplified using only 5 PCR cycles. The PCR product was eluted, digested with *MmeI* restriction enzyme and then ligated to a double-stranded DNA adapter. The ligated product was again purified and amplified using 15 cycles of PCR. The final PCR product was sequenced. The reads were processed for quality and then aligned to the transcriptome assembly of *M. truncatula* to identify potential miRNA targets using the SeqTar pipeline [29].

## Results and discussion

### The analyses of small RNA libraries

High-throughput sequencing has been used to identify miRNAs and their target mRNAs in plants [15, 30, 31]. To catalogue conserved and novel miRNAs in alfalfa, a total of eight small RNA libraries from the whole plants, roots and shoots of Altet-4, NECS-141 and NF08ALF06 genotypes were constructed and sequenced. After removal of the adapter sequences and low-quality reads, the total reads ranging between 11 to 42 million, and unique reads ranging between 1.8 to 8.5 million reads from these nine libraries were obtained (Table 1). However, the quality of the small RNA library generated from the shoots of NF08ALF06 did not meet the threshold criteria, therefore only NECS-141 and Altet-4 were used for the miRNA analyses of shoot tissues.

**Table 1:** The mapping of total and unique reads obtained from different small RNA libraries

	Altet-4 whole plants		NECS-141 whole plants		NF08ALF06 whole plants		Altet-4 Roots		NECS-141 Roots		NF08ALF06 Roots		Altet-4 Shoots		NCES-141 Shoots	
	Total reads	Unique reads	Total reads	Unique reads	Total reads	Unique reads	Total reads	Unique reads	Total reads	Unique reads	Total reads	Unique reads	Total reads	Unique reads	Total reads	Unique reads
cdna	6,858,719	336,266	6,197,308	430,819	8,142,985	493,929	9,352,477	276,276	19,665,339	732,738	8,930,063	352,167	17,117,689	909,491	10,943,411	669,572
ncRNAs	6,810,937	261,134	5,666,067	213,396	7,722,727	284,785	9,633,993	271,496	18,454,661	272,596	8,834,028	237,827	14,718,199	289,087	7,855,629	128,514
pre-miRBase	567,518	3182	943,976	3888	1,162,233	4005	147,326	2409	1,102,879	5780	426,327	3213	2,520,492	7051	3,840,771	6449
repeats	5,451,840	162,552	4,218,992	148,297	5,756,349	183,418	8,267,063	158,310	15,744,403	180,030	7,536,403	146,043	10,855,687	192,377	3,798,312	104,708
genome	8,951,430	1,142,594	9,878,838	2,398,705	11,387,413	2,053,582	11,557,742	784,140	29,143,549	5,078,322	11,588,832	1,488,546	28,744,231	5,246,027	29,192,098	5,834,731
total	12,008,892	2,343,120	11,645,217	3,348,188	15,733,102	3,739,163	14,377,336	1,860,736	33,335,201	6,947,622	14,378,859	2,708,737	42,196,888	8,564,218	34,441,313	7,748,996

Quantification of miRNA abundances between the genotypes and tissues was preceded by normalizing the expression levels of miRNA families to reads per ten million (RPTM). The normalized miRNA family read frequencies ranged between 1 to 552,267 RPTM for the whole plants, between 1 to 134,679 RPTM for the root samples, and 1 to 165,310 RPTM for the shoot samples (Table 2). The range of miRNA read frequencies varied slightly between the three genotypes. As expected, the most conserved miRNAs appeared to be the most abundantly expressed in all tissues and genotypes, with the exception of miR169, miR393, miR395 and miR172 which exhibited low abundances. Specifically, miR172 levels in roots and shoots of the three genotypes were extremely low and in most cases was below 20 RPTM (Table 2). The miRNA families with the lowest expression levels, and in some cases as low as 1 RPTM, were largely represented by the non-conserved miRNAs or miRNAs that have been reported exclusively from *M. truncatula* (miRBase) that include miR2601, miR2674, miR5207, miR5241, miR5243, miR5244, miR5255, miR5257, miR5269, miR5282, miR5289, miR5294, miR5296, miR5299, miR5561, miR5744, and miR7701 (Table 2). miR5207 is the only miRNA that was also reported from *Gossypium raimondii* (miRBase). The majority of the miRNA families identified are 21 nt long, although some cases including miR2601 and miR2603 were represented by 22 nucleotides. Further, a total of 23 miRNA families included between miR5267 to miR5299 were 24 nt long. The fact that these small RNAs were initially identified in *M. truncatula* (miRBase), and could be identified in several independent small RNA libraries from three different alfalfa genotypes (Table 2), suggests that these sequences and their associated processing are conserved between alfalfa and its close relative *M. truncatula*. However, their extremely low abundances coupled with their longer read lengths could also indicate that these may be 24-nt long siRNAs. Additional studies are needed to assess the precise nature of these small RNAs, i.e., miRNAs or siRNAs.

**Table 2:** Identified miRNA families and their frequencies (reads per ten million [RPTM]) in whole plants, roots and shoots of three alfalfa genotypes (miRNA-stars were marked in bold)

	Whole plants			Roots			Shoots	
	Altet-4	NECS-141	NF08ALF06	Altet-4	NECS-141	NF08ALF06	Altet-4	NECS-141
miR156-5p	4712	7243	6436	1001	3466	3145	19,808	47,306
**miR156-3p**	3262	4012	2992	545	755	548	4634	6420
miR159-3p	6315	11,050	8484	3910	23,465	10,549	61,929	103,370
miR160-5p	225	417	351	20	277	113	3505	8706
miR162-3p	140	229	292	194	454	361	533	517
miR164-5p	108	275	306	6	77	57	48	431
miR166-3p	336,905	552,267	534,054	34,634	111,596	134,679	101,118	131,196
**miR166-5p**	544	960	614	228	508	438	800	1216
miR167-5p	218	470	722	107	240	357	699	1389
**miR167-3p**	2	1	0	0	0	0	0	0
miR168-5p	1121	1980	1691	735	2960	1317	3460	5049
**miR168-3p**	672	691	768	182	443	194	5550	5638
miR169-5p	19	34	35	47	55	35	46	59
**miR169-3p**	7	12	5	6	18	7	2	2
miR171-3p	51	120	232	44	238	316	60	85
**miR171e-5p**	26	39	44	22	37	42	7	6
miR172-3p	62	138	240	0	1	1	2	3
**miR172-5p**	3	8	20	1	1	2	2	2
miR319-3p	1631	3689	2101	1607	6281	3323	4330	10,864
**miR319-5p**	46	72	74	3	20	14	129	559
miR390-5p	95	410	318	86	656	234	121	382
miR393-5p	11	24	34	4	8	10	22	45
miR395-3p	3	8	7	12	13	7	2	0
miR396-5p	12,185	21,926	22,411	2835	14,549	8121	39,236	58,336
**miR396-3p**	250	437	437	76	312	188	323	356
miR397-5p	57	28	15	37	16	11	94	61
**miR398a-5p**	19	16	25	0	2	1	4	3
miR398-3p	3814	3223	2272	2101	4086	3176	35,538	26,478
miR399-3p	17	11	11	25	26	13	62	43
miR408-3p	2656	1301	1096	977	737	570	6380	2866
**miR408-5p**	17	7	12	12	14	8	55	35
miR482-3p	28	27	49	18	19	45	41	105
**miR482-5p**	7	10	10	11	19	13	9	12
miR530-5p	2	7	8	0	1	1	2	4
miR1507–3	963	1789	1701	881	1596	1230	1778	3349
miR1510-5p	1959	4278	3520	523	3505	1429	12,496	34,705
**miR1510-3p**	96	151	167	52	118	63	256	617
miR2111	47	20	10	44	15	42	278	22
miR2118	5607	11,948	16,134	106	610	307	79,977	165,310
miR2199	95	15	42	21	18	30	156	13
miR2585	57	7	74	28	1	22	239	10
miR2587	0	6	9	0	10	10	13	28
miR2590	15	41	42	23	55	25	109	177
miR2592	393	1350	395	119	1612	268	1224	1742
miR2601-5p	0	0	0	0	0	0	1	1
miR2603-5p	0	8	1	1	1	1	5	24
miR2629-5p	2	5	4	1	3	7	2	5
miR2632-5p	0	1	0	0	0	0	1	18
miR2634-3p	5	3	7	6	4	15	9	6
miR2643-3p	1502	2689	2106	382	1462	948	9682	24,971
miR2651-3p	27	52	22	4	21	7	40	49
miR2661-5p	3	4	5	2	4	5	13	9
miR2666-3p	0	21	0	0	14	0	0	29
miR2674-3p	0	0	1	0	0	0	0	0
miR2678-3p	2	6	4	0	4	4	4	12
miR4414-3p	2	4	4	0	1	1	3	7
miR4414-5p	1	3	4	1	1	0	5	7
miR5037-5p	4	3	13	3	8	24	2	4
miR5204-3p	4	10	6	3	28	17	6	10
miR5205-5p	7	22	14	0	6	6	15	6
miR5207-5p	0	0	0	0	0	1	0	1
miR5208-3p	2	1	1	0	0	0	1	1
miR5208d-5p	0	0	1	0	1	0	1	1
miR5211-5p	432	85	23	559	71	41	292	59
miR5213-5p	801	836	887	181	891	829	1397	1379
miR5214-3p	63	155	153	97	414	452	153	201
miR5225-5p	4	2	8	3	1	8	1	1
miR5230-5p	1	2	1	0	1	0	6	1
miR5231-5p	10	7	7	3	11	1	43	69
miR5232-5p	67	253	419	56	503	417	602	3964
miR5237-3p	2	2	0	0	2	1	6	4
miR5238-5p	2	0	2	1	2	1	0	0
miR5239-5p	347	269	430	16	52	72	622	773
miR5241-3p	0	0	0	0	0	0	0	1
miR5243-3p	0	0	0	0	0	0	0	1
miR5244-3p	0	1	1	0	0	0	0	1
miR5248-5p	0	2	1	0	2	1	0	3
miR5255-3p	0	1	1	0	0	0	0	1
miR5257-5p	1	0	0	0	0	0	0	0
miR5261-3p	76	89	93	22	302	127	283	227
miR5266-5p	0	0	0	4	2	3	0	1
miR5267-5p	1	3	1	0	1	1	0	2
miR5269-3p	0	1	1	1	0	0	0	0
miR5271-5p	1	1	1	1	2	2	1	1
miR5272-5p	17	22	12	12	34	21	18	18
miR5273-3p	1	3	1	1	3	1	4	2
miR5277-3p	60	108	62	75	99	48	16	20
miR5279-5p	3	19	13	1	16	8	8	7
miR5281-3p	29	47	29	35	69	18	141	150
miR5282-3p	0	0	0	0	0	0	1	0
miR5284-3p	20	52	50	4	14	17	10	23
miR5285-5p	0	0	1	1	1	0	2	3
miR5286-3p	2	0	2	1	3	2	3	2
miR5287-3p	6	10	14	8	9	4	17	19
miR5289-3p	0	1	0	1	0	0	0	0
miR5290-3p	0	5	1	1	2	1	2	6
miR5291-3p	0	1	1	0	3	1	0	1
miR5292-3p	16	35	21	6	34	21	53	82
miR5294-3p	0	0	1	0	0	0	0	0
miR5295-3p	9	29	13	3	15	9	7	6
miR5296-3p	1	0	0	0	0	0	0	0
miR5297-3p	0	1	2	1	0	1	1	1
miR5298-3p	4	4	1	4	0	1	3	15
miR5299-3p	0	1	0	0	1	1	0	0
miR5558-5p	539	1938	1820	220	415	412	1103	1276
miR5559-5p	7	3	0	0	0	0	8	5
miR5561-3p	5	14	18	0	5	5	4	5
miR5561-5p	0	0	0	1	0	0	0	0
miR5743-5p	19	113	6	0	1	1	70	398
miR5744-5p	0	0	0	0	0	1	0	0
miR5745-3p	28	39	41	69	144	171	126	113
miR5752-3p	0	4	0	0	1	0	8	11
miR5754-5p	0	6	19	0	1	3	2	41
**miR7696-5p**	0	1	1	0	1	0	0	1
miR7696-3p	174	95	253	40	138	184	1173	255
miR7701-3p	0	1	0	0	0	0	0	0

### MicroRNA profiles in alfalfa plants, roots and shoots

A total of 100 known miRNA families were identified from the small RNA libraries of the three alfalfa genotypes (Table 2). Of these, 21 families were represented by the highly conserved miRNAs, whereas the remaining 79 families could be considered as *Medicago*-specific miRNA families. The identification of these 79 miRNA families in alfalfa was based on their expression in *M. truncatula* (miRbase), therefore, these are conserved at least between *M. truncatula* and alfalfa.

Among the highly conserved miRNA families, miR166 was the most highly expressed family in seven of the eight samples that were surveyed in this study. The only exception to this trend was observed in the shoots of NECS-141 in which the miR2118 family was the most abundant followed by the miR166 family. The miRNA families, miR396 and miR2118 represents the second and third most abundantly expressed in the whole plants, while miR159 and miR396 were the second and third most highly expressed miRNAs in roots. Several additional miRNA families including miR398, miR160, miR168, miR319, miR408, miR1510 and miR2643 were also highly expressed but miR169, miR171, miR393, miR397 and miR395 were expressed at relatively very low levels (Table 2). On the other hand, miR159, miR156, miR319, miR398 miR1507 and miR1510 were highly expressed but miR164, miR169, miR172, miR393, miR397, miR399 and miR482 were expressed at very low levels in roots of these genotypes. Interestingly, miR160 was not sequenced from the roots of three alfalfa genotypes.

Overall, the conserved miRNA families such as the miR156, miR159, miR166, miR168, miR319, miR396, miR398 and miR408 were more highly expressed in the plants, roots and shoots of all three alfalfa genotypes. Among the legume-specific families, miR1507, miR1510, miR2118, miR2592, miR2643, miR5213, miR5232, miR5558 and miR7696 (Table 2) were also abundant in all tissues of alfalfa genotypes. Conversely, some conserved miRNA families represented by miR169 and miR393 recorded very low abundances in all samples. Other notable differences between roots and shoots include relatively low expression levels of miR160, miR167, and miR408 in roots compared to the shoots of alfalfa genotypes (Table 2).

Several miRNA families including miR482, miR1507, miR2118, miR4416 are conserved in *M. truncatula*, soybean, chickpea (miRBase). These miRNA families are known to regulate NBS-LRR genes that are involved in pathogen resistance. The miRNA-guided cleavage on the NBS-LRR genes initiates the generation of phasiRNAs [16, 18, 32]. In alfalfa, miR482, miR1507 and miR2118 were detected in all three tissues (Table 2), but not miR4416. Both miR2118 and miR1507 families were more abundantly expressed in all tissues and genotypes compared with miR482 family. Remarkably, miR2118 was the top most highly expressed miRNA family in shoots of NECS-141. By contrast, miR2118 levels were very low in roots of three alfalfa genotypes. On the other hand, miR1507 family displayed approximately similar levels in three tissues of alfalfa genotypes.

The miRNA-star sequences corresponding to the 12 of the 21 highly conserved miRNA families were also recovered from almost all libraries (Table 2). Additionally, miRNA-stars for the miR1510, miR4414, miR5208, and miR7696 were also detected. Furthermore, the miRNA-star expression levels for miR156, miR166 and miR168 were very high (Table 2). Intriguingly, like miR168, miR168 star levels differed greatly between different tissue. In shoots of NECS-141, miR168 star levels were slightly more than that of miR168, while both in whole plants and roots, the star levels were approximately half of the levels of miR168.

### miRNA diversity in alfalfa compared with other legumes

Several miRNA families are specifically reported from the leguminous plants such as the *M. truncatula, Glycine max*, *Lotus japonicus*, *Phaseolus vulgaris*, *Cicer arietinum*, *Vigna unguiculata* and *Acacia auriculiformis* [14, 16, 18, 19, 32, 33]. These lineage-specific miRNAs include miR1507, miR1508, miR1509, miR1510, miR1512, miR1514, miR1520, miR1521, miR2118, miR2086, miR2109, miR2199, miR4414, miR5213, miR5232, and miR5234 among others (miRBase). The majority of these were reported from *M. truncatula* and soybean, since these legume species have been the subject of multiple studies exploring small RNAs. Most of these legume-specific miRNAs were also identified in alfalfa and a few of them including miR1507, miR1510, miR2118, miR2592, miR2643, miR5211, miR5213, miR5214, miR5232, miR5239, miR5277, miR5558, and miR7696 were specifically highly expressed in all three genotypes (Table 2).

### Identification of novel miRNAs from alfalfa

**Fig. 1:**
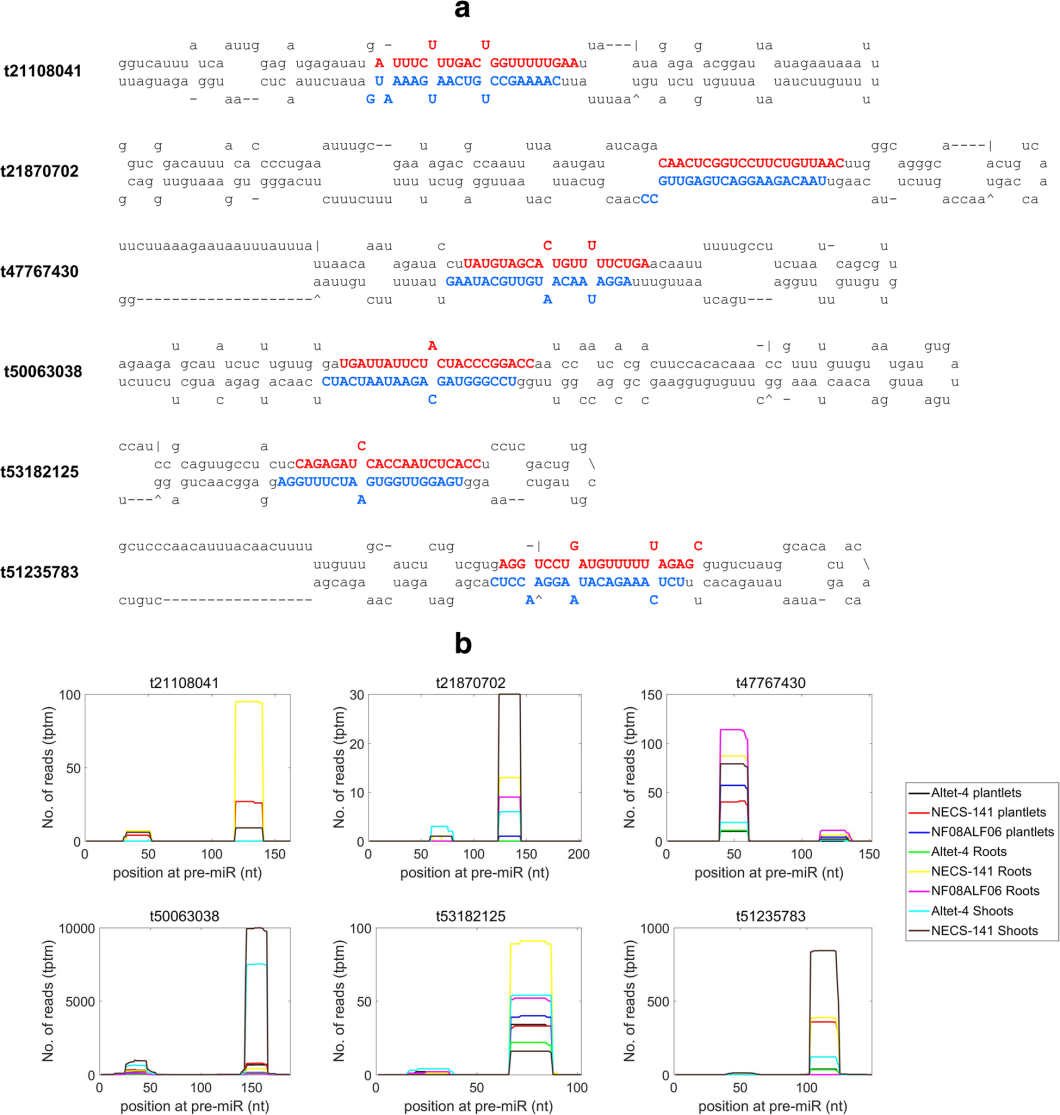
The predicted foldback structures using the novel miRNA precursor sequences. **a** The fold-back structures for six novel miRNAs. **b** The distribution of small RNA reads on the precursors of the novel miRNAs depicted in Fig. 1a

The sequencing of the small RNAs from multiple tissues of three different alfalfa genotypes would allow us to identify the novel miRNAs more confidently. Novel miRNA identification was dependent on sequencing of the miRNA complementary strand (miRNA-star) coupled with the predictable fold back structure for the primary miRNA transcript. Because a stable assembly of the tetraploid alfalfa genome sequence is not available, the small RNAs were mapped to the *M. truncatula genome*. Mapping of the small RNAs from the three alfalfa genotypes onto the *M. truncatula* genome enabled the identification of novel miRNAs more confidently because they have been sequenced from *M. sativa* and mapped on to the *M. truncatula*, suggesting their conservation between *M. sativa* and *M. truncatula*. Moreover, the novel miRNA identification in this study is more robust as it includes sequencing of these small RNAs from three different genotypes. We have identified a total of 17 novel miRNAs which have been sequenced from all of the three genotypes (Table 3 and Fig. 1). Among these, t50582913 was highly expressed followed by t50063038. In roots, t50582913 was highly expressed in NECS-141 and Altet-4 but not in NF08ALF06. In shoots, t50063038 was highly expressed followed by the t50582913 and t51235783.

**Table 3:** Identified novel miRNAs based on sequencing both 5′ and 3′ reads and the most abundant ones that is marked in bold denotes potential novel miRNA based on their greater abundances

miR-5p	miR-5p_seq	miR-3p	miR-3p_seq	Altet-4 Plants	NECS-141 Plants	NF08ALF06 Plants	Altet-4 Roots	NECS-141 Roots	NF08ALF06 Roots	Altet-4 Shoots	NECS-141 Shoots
t61680599	UUUCUUUGACUGGUUUUUGAAU	**t21108041**	CAAAAGCCUGUCAAUGAAAAUG	0	31	0	0	312	0	0	32
t46402976	UAGCAUCAAGCGUCGCGUCGAU	**t28372577**	CGACCCGAGGCUUAUGCGAUC	115	97	145	81	479	229	335	315
t59820880	UUGGCAGAAUCACGGUGUGCC	**t29809748**	CGGUGGCAUCGUGAUUUUGAC	0	6	25	1	6	8	1	47
t21870702	CAACUCGGUCCUUCUGUUAAC	**t44359413**	UAACAGAAGGACUGAGUUGCC	0	11	3	1	41	12	24	103
t62603216	UUUUCAAGUUGGUCCCUUACG	**t44814359**	UAAGGGACCAACUUGAAAACU	77	178	196	7	240	107	529	899
t8901469	ACCUGGAGACAGAGAUGCAAU	**t45832108**	UACGUCUCUGUCUUUCGGGUUG	1	55	28	2	222	28	6	247
t12927907	AGGAUAACAAUGUUGCAUAAG	**t47767430**	UAUGUAGCACUGUUUUUCUGA	13	43	85	14	273	147	83	262
**t63076572**	UUUUUAGAUACAUUGAAUAAU	t47960370	UAUUCAAUGUAUCUAAAAAG	10	10	14	4	40	2	208	177
t53501433	UGAUUAUUCUACUACCCGGACC	**t50063038**	UCCGGGUAGCAGAAUAAUCAUC	350	371	78	45	371	18	17,057	20,494
t12458129	AGCGGUUGGUACAAUGCAAUAu	**t50582913**	UCGCCUUGUACCAACCUACUGC	544	915	0	123	1148	0	881	1453
t40560414	GGUCCUGAUGUUUUUUAGAGC	**t51235783**	UCUCAAAGACAUAAGGAACCUC	19	281	0	24	762	0	269	1655
t55270980	UGUCUUUAGCUUCCGAAACAa	**t55621674**	UGUUCCGGUAGAUGAAGUCAC	4	4	0	2	23	0	24	40
t14211567	AGUUAAUUGUGUUGCAUGAGUU	**t57726911**	UUCAGCAACAUGAGUUAACUCA	17	26	60	3	48	22	42	50
**t8194733**	ACAUUUUAGAUUGUUGAGGAA	t27568341	CCUCAAUUAUCUAUUAUGUUU	0	0	3	0	3	6	6	0
**t62313817**	UUUGUUAAACAUUUGUUUCC	t311560	AAAACAAAUGUUUAGCUAAG	0	6	0	0	15	1	0	12
t55268921	UGUCUUGGUUUCAAAAAGAAGu	**t52170136**	UCUUUUUGCAAACCAACUCAAU	4	19	13	1	29	4	9	56
**t51870988**	UCUUAUUUUCGACAUUGCAAAG	t59475847	UUGCAGGUCGAGAAUAAAAUG	19	99	71	1	9	1	353	1072

### Identification of miRNA targets in alfalfa

Although the alfalfa is one of the important legumes agronomically, the genome sequencing and annotations are not available so far. Due to this, studies have utilized the well-studied and closely related *M. truncatula* genome annotations as a model for alfalfa studies. The nucleotide identity for some genes was greater than 97% between *M. sativa* and *M. truncatula* [34]). Thus, using *M. truncatula* transcript annotations can facilitate identification of miRNA targets in alfalfa. We used SeqTar algorithm (Zheng et al., 2012) to identify miRNA targets by allowing a maximum of 4 mismatches between miRNAs and their potential target transcripts.

Previous studies have revealed that conserved miRNAs are strongly associated with the regulation of genes that encode transcription factors [35]. These transcription factors in turn regulate key developmental processes and pathways in plants. Degradome sequencing has been very effective in identifying plant miRNA targets. Besides identifying the conserved targets, this approach can also identify non-conserved targets for the conserved miRNAs [28, 36, 37]. Degradome sequencing was used in this study to identify the cleaved mRNA fragments corresponding to the miRNA recognition sites in all three alfalfa genotypes. Approximately 30 million degradome reads were obtained from the transcripts of each of the alfalfa genotypes (Table 4) and these reads were analysed using SeqTar program. In total, we have identified 69 targets for 31 miRNA families that included 16 highly conserved families (Table 5). With respect to the conserved miRNAs, 33 targets for 16 conserved miRNA families were identified (Table 5). The known targets for miR162, miR165/166, miR398 and miR399 families were not identified in this study. Although miR165/166 family is the most abundantly expressed as scored from their read frequencies in almost all libraries but the cleaved fragments from the HD-Zip target transcripts were not recovered from degradome libraries of alfalfa genotypes.

**Table 4:** Mapping of the reads obtained from the degradome libraries

Database	Altet-4		NECS-141		NF08ALF06	
	Total reads	Unique reads	Total reads	Unique reads	Total reads	Unique reads
*M. truncatula* genome	852,790	487,582	1,541,055	791,294	3,091,832	1,021230
*M. sativa* genome	1,488,681	957,866	2,691,763	1,435,659	4,591,130	1,877,041
Cds	770,970	426,278	1,436,059	727,330	2,928,098	933,425
ncRNA	231,076	22,907	186,813	18,014	1,305,681	36,585
Repeats	171,358	16,804	116,741	16,759	636,675	23,958
Pre-miRBase	34,631	837	35,979	1045	50,136	1192
Total	28,674,678	2,286,693	30,573,270	3,137,327	30,812,606	3,885,547

**Table 5:** miRNA targets identified in the degradome libraries generated from three alfalfa genotypes. #Mis. is number of mismatches on the miRNA complementary site; Valid reads is Reads corresponding to the expected cleavage site; Total reads is Total reads mapped to the cDNA of the gene; Percent is Percent reads at the expected cleavage site

genotypes	miRNA id#	Target gene	#Mis.	Valid reads	Total reads	Percent	Target gene annotation
Altet-4	miR156e	Medtr7g028740.2	0	4	23	17.4	squamosa promoter-binding-like protein
Altet-4	miR156a	Medtr7g444860.1	0	2	28	7.1	squamosa promoter-binding-like protein
Altet-4	miR156a	Medtr3g099080.1	0	1	3	33.3	squamosa promoter-binding 13A-like protein
Altet-4	miR159b	Medtr8g042410.1	2.5	1	16	6.3	MYB transcription factor
Altet-4	miR160c	Medtr2g094570.3	1	4	21	19.1	auxin response factor 1
Altet-4	miR164d	Medtr2g064470.1	1	2	34	5.9	NAC transcription factor-like protein
Altet-4	miR164d	Medtr8g058330.1	2	5	49	10.2	protein transporter Sec61 subunit alpha-like protein
Altet-4	miR167b-5p	Medtr8g079492.3	4	4	62	6.5	auxin response factor 2
Altet-4	miR169e-5p	Medtr2g099490.2	2	1	20	5	CCAAT-binding transcription factor
Altet-4	miR171f	Medtr0092s0100.2	1.5	5	24	20.8	GRAS family transcription regulator
Altet-4	miR172a	Medtr4g094868.3	1	1	13	7.7	AP2 domain transcription factor
Altet-4	miR172a	Medtr5g016810.2	1	1	18	5.6	AP2 domain transcription factor
Altet-4	miR172a	Medtr2g093060.3	0	4	17	23.5	AP2-like ethylene-responsive transcription factor
Altet-4	miR319d-3p	Medtr2g078200.1	3	2	34	5.9	TCP family transcription factor
Altet-4	miR319d-3p	Medtr8g463380.1	3	2	7	28.6	TCP family transcription factor
Altet-4	miR393a	Medtr1g088950.1	1	11	83	13.3	transport inhibitor response-like protein
Altet-4	miR393a	Medtr7g083610.1	2	38	134	28.4	transport inhibitor response 1 protein
Altet-4	miR395j	Medtr1g102550.1	1	1	76	1.3	ATP sulfurylase
Altet-4	miR396b-5p	Medtr1g017490.2	3	47	100	47	growth-regulating factor
Altet-4	miR396b-5p	Medtr2g041430.3	3	5	12	41.7	growth-regulating factor-like protein
Altet-4	miR396b-5p	Medtr5g027030.1	3	5	15	33.3	growth-regulating factor
Altet-4	miR396a-5p	Medtr3g052060.1	2	1	1	100	hypothetical protein
Altet-4	miR398c	Medtr4g114870.1	3	8	23	34.8	plastocyanin-like domain protein
Altet-4	miR398a-3p	Medtr8g064810.1	3	5	36	13.9	protein disulfide isomerase (PDI)-like protein
Altet-4	miR408-3p	Medtr8g089110.1	3	3	9	33.3	basic blue-like protein
Altet-4	miR408-3p	Medtr8g007020.1	3.5	5	73	6.9	plastocyanin-like domain protein
Altet-4	miR408-3p	Medtr8g007035.1	3.5	5	123	4.1	plastocyanin-like domain protein
Altet-4	miR408-5p	Medtr3g074830.1	3.5	2	442	0.5	phosphate-responsive 1 family protein
Altet-4	miR1510a-5p	Medtr2g012770.1	1	1	5	20	disease resistance protein (TIR-NBS-LRR class)
Altet-4	miR2199	Medtr7g080780.2	2	2	8	25	helix loop helix DNA-binding domain protein
Altet-4	miR2643a	Medtr3g010590.1	1	1	15	6.7	F-box protein interaction domain protein
Altet-4	miR2643a	Medtr6g053240.1	3	2	4	50	F-box protein interaction domain protein
Altet-4	miR4414a-5p	Medtr3g117120.1	4	3	84	3.6	BZIP transcription factor bZIP124
Altet-4	miR5213-5p	Medtr6g084370.1	2	1	2	50	disease resistance protein (TIR-NBS-LRR class)
Altet-4	miR5213-5p	Medtr6g088245.1	3	1	5	20	disease resistance protein (TIR-NBS-LRR class)
Altet-4	miR5239	Medtr3g018680.1	3	1	5	20	F-box/RNI superfamily protein, putative
Altet-4	miR5561-3p	Medtr2g045295.1	3	1	4	25	hypothetical protein
Altet-4	miR5752b	Medtr8g066820.1	4	9	423	2.1	PLATZ transcription factor family protein |
Altet-4	miR7696a-5p	Medtr1g072130.1	3	2	27	7.4	PHD finger protein, putative
Altet-4	miR7696c-3p	Medtr3g081480.1	3	2	21	9.5	endoplasmic reticulum vesicle transporter
Altet-4	miR7696d-5p	Medtr3g112250.1	3.5	8	36	22.2	hypothetical protein
Altet-4	miR7696c-3p	Medtr4g011600.2	3.5	1	26	3.9	sulfate transporter-like protein
Altet-4	miR7696c-3p	Medtr7g085650.4	3.5	1	6	16.7	sulfate adenylyltransferase subunit 1/adenylylsulfate kinase
Altet-4	miR7701-3p	Medtr6g011380.2	2	1	137	0.7	SPFH/band 7/PHB domain membrane-associated family protein
NF08ALF06	miR156e	Medtr7g028740.2	0	14	36	38.9	squamosa promoter-binding-like protein
NF08ALF06	miR156a	Medtr7g444860.1	0	1	144	0.7	squamosa promoter-binding-like protein
NF08ALF06	miR156h-3p	Medtr7g091370.1	3	1	11	9.1	heat shock transcription factor
NF08ALF06	miR159b	Medtr8g042410.1	2.5	4	30	13.3	MYB transcription factor
NF08ALF06	miR160c	Medtr2g094570.3	1	8	46	17.4	auxin response factor 1
NF08ALF06	miR160d	Medtr1g064430.2	0.5	3	24	12.5	auxin response factor 1
NF08ALF06	miR160d	Medtr3g073420.1	0.5	2	17	11.8	auxin response factor, putative
NF08ALF06	miR164d	Medtr2g064470.1	1	41	151	27.2	NAC transcription factor-like protein
NF08ALF06	miR164d	Medtr8g058330.1	2	5	115	4.4	protein transporter Sec61 subunit alpha-like protein
NF08ALF06	miR167b-5p	Medtr8g079492.3	4	9	133	6.8	auxin response factor 2
NF08ALF06	miR167a	Medtr4g076020.1	3.5	5	77	6.5	GRAS family transcription factor
NF08ALF06	miR171f	Medtr0092s0100.2	1.5	60	115	52.2	GRAS family transcription regulator
NF08ALF06	miR172a	Medtr4g094868.3	1	1	45	2.2	AP2 domain transcription factor
NF08ALF06	miR172a	Medtr5g016810.2	1	1	84	1.2	AP2 domain transcription factor
NF08ALF06	miR172a	Medtr2g093060.3	0	4	35	11.4	AP2-like ethylene-responsive transcription factor
NF08ALF06	miR172a	Medtr4g061200.4	1	1	28	3.6	AP2-like ethylene-responsive transcription factor
NF08ALF06	miR172a	Medtr7g100590.1	1	2	17	11.8	AP2 domain transcription factor
NF08ALF06	miR319d-3p	Medtr2g078200.1	3	2	126	1.6	TCP family transcription factor
NF08ALF06	miR319d-3p	Medtr8g463380.1	3	2	48	4.2	TCP family transcription factor
NF08ALF06	miR393a	Medtr1g088950.1	1	54	268	20.2	transport inhibitor response-like protein
NF08ALF06	miR393a	Medtr7g083610.1	2	472	771	61.2	transport inhibitor response 1 protein
NF08ALF06	miR393a	Medtr8g098695.2	4	1	46	2.2	transport inhibitor response 1 protein
NF08ALF06	miR396b-5p	Medtr1g017490.2	3	423	742	57	growth-regulating factor
NF08ALF06	miR396b-5p	Medtr2g041430.3	3	30	75	40	growth-regulating factor-like protein
NF08ALF06	miR396b-5p	Medtr5g027030.1	3	10	42	23.8	growth-regulating factor
NF08ALF06	miR396a-5p	Medtr3g011560.1	3	1	3	33.3	TNP1
NF08ALF06	miR396a-5p	Medtr3g052060.1	2	3	11	27.3	hypothetical protein
NF08ALF06	miR396a-5p	Medtr8g017000.1	3	1	2	50	Ulp1 protease family, carboxy-terminal domain protein
NF08ALF06	miR398c	Medtr4g114870.1	3	14	49	28.6	plastocyanin-like domain protein
NF08ALF06	miR398a-3p	Medtr8g064810.1	3	8	44	18.2	protein disulfide isomerase (PDI)-like protein
NF08ALF06	miR408-3p	Medtr8g089110.1	3	8	34	23.5	basic blue-like protein
NF08ALF06	miR408-3p	Medtr8g007020.1	3.5	10	375	2.7	plastocyanin-like domain protein
NF08ALF06	miR408-3p	Medtr8g007035.1	3.5	10	675	1.5	plastocyanin-like domain protein
NF08ALF06	miR408-5p	Medtr3g074830.1	3.5	27	948	2.9	phosphate-responsive 1 family protein
NF08ALF06	miR482-5p	Medtr1g064430.2	3.5	1	24	4.2	auxin response factor 1
NF08ALF06	miR530	Medtr3g072110.1	2.5	3	102	2.9	transmembrane amino acid transporter family protein
NF08ALF06	miR1507–3p	Medtr8g036195.1	2	4	9	44.4	NBS-LRR type disease resistance protein
NF08ALF06	miR1510a-5p	Medtr7g108860.4	3.5	21	1061	2	CS domain protein
NF08ALF06	miR2199	Medtr7g080780.2	2	1	26	3.9	helix loop helix DNA-binding domain protein
NF08ALF06	miR2643a	Medtr6g053240.1	3	25	33	75.8	F-box protein interaction domain protein
NF08ALF06	miR4414a-5p	Medtr3g117120.1	4	8	260	3.1	BZIP transcription factor bZIP124
NF08ALF06	miR5037c	Medtr4g070550.1	3	2	44	4.6	F-box protein interaction domain protein
NF08ALF06	miR5213-5p	Medtr4g014580.1	1.5	3	31	9.7	TIR-NBS-LRR class disease resistance protein
NF08ALF06	miR5238	Medtr3g077740.2	2.5	1	259	0.4	pantothenate kinase
NF08ALF06	miR5239	Medtr3g018680.1	3	4	43	9.3	F-box/RNI superfamily protein, putative
NF08ALF06	miR5561-3p	Medtr2g045295.1	3	1	12	8.3	hypothetical protein
NF08ALF06	miR5752a	Medtr8g066820.1	4	13	936	1.4	PLATZ transcription factor family protein
NF08ALF06	miR7696a-5p	Medtr1g072130.1	3	4	259	1.5	PHD finger protein, putative
NF08ALF06	miR7696c-3p	Medtr3g081480.1	3	2	46	4.4	endoplasmic reticulum vesicle transporter
NF08ALF06	miR7696c-5p	Medtr7g076830.1	3	3	103	2.9	DEAD-box ATP-dependent RNA helicase-like protein
NF08ALF06	miR7696d-5p	Medtr3g112250.1	3.5	5	30	16.7	hypothetical protein
NF08ALF06	miR7696c-3p	Medtr4g011600.2	3.5	1	103	1	sulfate transporter-like protein
NF08ALF06	miR7696c-3p	Medtr7g085650.4	3.5	2	10	20	sulfate adenylyltransferase subunit 1/adenylylsulfate kinase
NF08ALF06	miR7701-3p	Medtr3g108910.1	2.5	2	375	0.5	hypothetical protein
NF08ALF06	miR7701-3p	Medtr6g011380.2	2	2	86	2.3	SPFH/band 7/PHB domain membrane-associated family protein
NCES-141	miR156e	Medtr7g028740.2	0	18	46	39.1	squamosa promoter-binding-like protein
NCES-141	miR156a	Medtr7g444860.1	0	4	101	4	squamosa promoter-binding-like protein
NCES-141	miR156a	Medtr8g096780.1	0	1	11	9.1	squamosa promoter-binding 13A-like protein
NCES-141	miR156a	Medtr3g085180.1	1	1	2	50	squamosa promoter-binding-like protein
NCES-141	miR156h-3p	Medtr7g091370.1	3	2	5	40	heat shock transcription factor
NCES-141	miR159b	Medtr8g042410.1	2.5	3	36	8.3	MYB transcription factor
NCES-141	miR160c	Medtr2g094570.3	1	12	37	32.4	auxin response factor 1
NCES-141	miR164d	Medtr2g064470.1	1	33	100	33	NAC transcription factor-like protein
NCES-141	miR164d	Medtr8g058330.1	2	14	119	11.8	protein transporter Sec61 subunit alpha-like protein
NCES-141	miR167b-5p	Medtr8g079492.3	4	10	101	9.9	auxin response factor 2
NCES-141	miR167a	Medtr4g076020.1	3.5	4	45	8.9	GRAS family transcription factor
NCES-141	miR167b-3p	Medtr4g124900.2	3.5	1	154	0.7	auxin response factor 2
NCES-141	miR168a	Medtr6g477980.2	4	2	245	0.8	argonaute protein 1A
NCES-141	miR171f	Medtr0092s0100.2	1.5	36	70	51.4	GRAS family transcription regulator
NCES-141	miR172a	Medtr4g094868.3	1	2	50	4	AP2 domain transcription factor
NCES-141	miR172a	Medtr5g016810.2	1	2	56	3.6	AP2 domain transcription factor
NCES-141	miR172a	Medtr2g093060.3	0	1	19	5.3	AP2-like ethylene-responsive transcription factor
NCES-141	miR172a	Medtr4g061200.4	1	3	32	9.4	AP2-like ethylene-responsive transcription factor
NCES-141	miR319d-3p	Medtr2g078200.1	3	1	55	1.8	TCP family transcription factor
NCES-141	miR319d-3p	Medtr8g463380.1	3	1	26	3.9	TCP family transcription factor
NCES-141	miR393a	Medtr1g088950.1	1	38	222	17.1	transport inhibitor response-like protein
NCES-141	miR393a	Medtr7g083610.1	2	337	539	62.5	transport inhibitor response 1 protein
NCES-141	miR395j	Medtr1g102550.1	1	1	163	0.6	ATP sulfurylase
NCES-141	miR396b-5p	Medtr1g017490.2	3	201	352	57.1	growth-regulating factor
NCES-141	miR396b-5p	Medtr5g027030.1	3	6	16	37.5	growth-regulating factor
NCES-141	miR396b-5p	Medtr8g020560.1	3	1	7	14.3	growth-regulating factor-like protein
NCES-141	miR396a-5p	Medtr3g011560.1	3	1	1	100	TNP1
NCES-141	miR396a-5p	Medtr8g017000.1	3	1	1	100	Ulp1 protease family, carboxy-terminal domain protein
NCES-141	miR397-5p	Medtr7g062310.1	1.5	2	4	50	laccase/diphenol oxidase family protein
NCES-141	miR398c	Medtr4g114870.1	3	8	21	38.1	plastocyanin-like domain protein
NCES-141	miR398a-3p	Medtr8g064810.1	3	47	89	52.8	protein disulfide isomerase (PDI)-like protein
NCES-141	miR398c	Medtr5g089180.1	3	4	19	21.1	hypothetical protein
NCES-141	miR408-3p	Medtr8g089110.1	3	9	18	50	basic blue-like protein
NCES-141	miR408-3p	Medtr8g007020.1	3.5	7	209	3.4	plastocyanin-like domain protein
NCES-141	miR408-3p	Medtr8g007035.1	3.5	8	381	2.1	plastocyanin-like domain protein
NCES-141	miR408-5p	Medtr3g074830.1	3.5	14	703	2	phosphate-responsive 1 family protein
NCES-141	miR482-3p	Medtr5g027900.1	2.5	1	19	5.3	disease resistance protein (CC-NBS-LRR class) family protein
NCES-141	miR530	Medtr3g072110.1	2.5	1	119	0.8	transmembrane amino acid transporter family protein
NCES-141	miR1510a-5p	Medtr7g108860.4	3.5	17	746	2.3	CS domain protein
NCES-141	miR2643a	Medtr3g010620.1	1	2	72	2.8	F-box protein interaction domain protein
NCES-141	miR4414a-5p	Medtr3g117120.1	4	2	134	1.5	BZIP transcription factor bZIP124
NCES-141	miR5037c	Medtr4g070550.1	3	1	36	2.8	F-box protein interaction domain protein
NCES-141	miR5213-5p	Medtr6g084370.1	2	1	5	20	disease resistance protein (TIR-NBS-LRR class)
NCES-141	miR5213-5p	Medtr4g014580.1	1.5	1	18	5.6	TIR-NBS-LRR class disease resistance protein
NCES-141	miR5213-5p	Medtr6g088245.1	3	1	7	14.3	disease resistance protein (TIR-NBS-LRR class)
NCES-141	miR5238	Medtr3g077740.2	2.5	1	151	0.7	pantothenate kinase
NCES-141	miR5561-3p	Medtr2g045295.1	3	1	9	11.1	hypothetical protein
NCES-141	miR5752b	Medtr8g066820.1	4	8	765	1.1	PLATZ transcription factor family protein
NCES-141	miR7696a-5p	Medtr1g072130.1	3	2	135	1.5	PHD finger protein, putative
NCES-141	miR7696c-5p	Medtr7g076830.1	3	5	78	6.4	DEAD-box ATP-dependent RNA helicase-like protein
NCES-141	miR7696d-5p	Medtr3g112250.1	3.5	9	44	20.5	hypothetical protein
NCES-141	miR7696c-3p	Medtr4g011600.2	3.5	1	124	0.8	sulfate transporter-like protein
NCES-141	miR7696c-3p	Medtr7g085650.4	3.5	1	10	10	sulfate adenylyltransferase subunit 1/adenylylsulfate kinase
NCES-141	miR7701-3p	Medtr3g108910.1	2.5	2	444	0.5	hypothetical protein

The identified miRNA targets in all three genotypes include mainly transcription factors. Specifically, five members of the squamosa promoter-binding-like protein (SPL) targeted by the miR156 family, five members of the auxin response factors targeted by both miR160 and miR167 families, five members of the apetala2 (AP2)-domain containing transcription factors, four members of the growth-regulating factor (GRFs) family targeted by miR396, two members of the TCP family transcription factors targeted by miR319, and, a NAC domain-containing transcription factor-like protein (NAC) targeted by miR164 [35]. Additionally, transcripts encoding Argonaute targeted by miR168, laccase targeted by miR397, and three plantacyanin containing proteins targeted by miR408 were also identified. Although evidence indicates that that miR398 targets Cu/Zn superoxide dismutases and a copper chaperone for the superoxide dismutases (CCS) in plants [28, 38] these relationships were not apparent in the data from this study. On the other hand, we have identified three potentially non-conserved targets (plastocyanin, protein disulphide isomerase and a hypothetical protein) for miR398 in three alfalfa genotypes. In addition to the GRFs, our analyses revealed potential non-conserved targets for miR396 including TNP1, Ulp1 protease and hypothetical proteins (Table 5).

The analyses of legume-specific miRNAs and their targets have revealed an interesting miRNA: target networks between the miRNAs and the NBS-LRR genes [14, 16, 18, 32]. In this study, we identified NBS-LRR disease resistance genes as targets for four different miRNA families including miR482, miR1507, miR1510 and miR5213 in alfalfa (Table 5).

Degradome analyses has also been utilized to identify potential targets for several non-conserved miRNAs or miRNAs that are present only in closely related species such as the *M. truncatula*. To increase the confidence in identification of targets for the non-conserved miRNAs that are usually expressed at low abundances and the cleavage frequencies on those targets are relatively low, we considered as ‘targets’ only those for which the cleavages were detected at least in two of the three alfalfa genotypes. The transcripts for Medtr6g053240.1 (F-box protein interaction domain protein) had a cleavage frequency of approximately 75% and were targeted by the miR2643 in NF08ALF06 genotype. Additionally, two other F-box protein interaction domain protein genes were also identified as targets for miR2643 in alfalfa genotypes (Table 5). These results suggest that the F-box protein interaction domain protein family are regulated by this potential legume-specific miRNA. Another notable observation is that 6 different genes identified as potential targets for miR7696, and the cleavage frequency of a particular target gene (hypothetical protein, Medtr3g112250.1) was more abundant in all three alfalfa genotypes (Table 5).

Because some of the miRNA-stars are also highly expressed, we scrutinized the degradome reads for potential cleavages on the transcripts that are complementary to the miRNA-stars. This analysis has identified potential targets for at least four conserved miRNAs. Specifically, miR156-star targets a heat shock transcription factor, miR164-star targets a protein transporter Sec61 subunit alpha-like protein, miR167-star targets a GRAS family transcription factor, and, miR482-star targets an auxin response factor 1 in alfalfa (Table 5).

The confirmed targets of conserved miRNAs are known to regulate diverse developmental processes in the lifecycle of plants. For example, the SPL transcription factors (target of miR156) which regulate the transition from juvenile to adult phase of the life cycle in land plants [39]. Auxin receptors (TIR1 proteins) and ARFs targeted by miR393 and miR160, miR167, are components of the auxin signalling pathway that regulates several aspects of plant growth and development. The roles of NAC factors (targeted by miR164) include shoot meristem initiation and later root formation in Arabidopsis [40, 41]. Similarly, TCP family transcription factors have several different roles including regulating leaf morphogenesis [42, 43]. In Arabidopsis, seven out of nine GRFs are known targets for miR396 [44], and we have identified four GRFs as targets for miR396 in alfalfa (Table 5). By interacting with its coactivators called GRF-interacting factors (GIFs), this regulatory network (miR396-GRFs-GIFs) regulate leaf size, leaf growth and senescence in Arabidopsis [44]. The known targets for miR397 include laccase, which is involved in oxidative polymerization of lignin in plants [45]. Similarly, miR408 is targeting a family of plantacyanins, which could function in shuttling electron-transfer between proteins [46, 47].

The miR398 family is known to target CSDs and a copper chaperone for superoxide dismutase (CCS) genes in plants [28, 38]. In this study, we have identified plastocyanin-domain like proteins (plastocyanin is an essential electron carrier which shuttles the electrons between cytochrome *b*_6_*f* and PS I) represents a novel target for miR398. Protein disulphide isomerase (PDI) is a member of a family of dithiol/disulfide oxidoreductases, the thioredoxin superfamily, which functions in the formation of disulphide linkage between the cysteine residues for proper protein folding [48]. Our degradome analyses confirms that PDI represents a novel target for miR398 in alfalfa (Table 5). The other confirmed miRNA target transcripts include Leucine rich repeat resistance (LRR) proteins (TIR-NBS-LRR and CC-NBS-LRR) that play important roles in plant pathogen recognition and activation of plant innate immune responses [14, 16, 18, 32]. Yet another interesting target include the F-box protein interaction domain proteins that are regulated by miR2643, one of the very abundantly expressed miRNA in alfalfa.

## Conclusions

The analyses of small RNA libraries from the whole plants, shoots and roots resulted in the identification of 100 miRNA families that included highly conserved miRNAs as well as miRNAs that are at least conserved between *M. truncatula* and alfalfa. The conserved miRNA profiles share some similarities and a few differences between genotypes and types of tissues (roots and shoots). The tissue-specific profiles were used to identify miRNAs that are highly abundant as well as those miRNAs that are expressed at low levels. Additionally, 17 novel miRNAs with varying levels of expression were also identified in alfalfa. The present study also reports identification of 69 targets for 31 miRNA families. In addition to the conserved targets for conserved miRNAs, a few non-conserved targets such as the PDI for miR398 were confirmed. Similarly, miR2643 is targeting three transcripts encoding F-box protein interaction domain containing proteins in alfalfa. In summary, the results from this study have increased our understanding of miRNAs and miRNA-mediated gene regulation in alfalfa that could result in potential tangible targets for practical applications in alfalfa and related legume species to increase biomass yield and address abiotic and biotic limitations to agricultural productivity.
